# An NADPH sensor that regulates cell ferroptosis

**DOI:** 10.1186/s12967-022-03658-3

**Published:** 2022-10-20

**Authors:** Guixing Kuang, Weidong Wang, Dan Xiong, Chong Zeng

**Affiliations:** 1 Department of Clinical Laboratory, Guangzhou Panyu Sixth People’s Hospital, 511442 Guangzhou, China; 2 Department of Hepatobiliary Surgery, Shunde Hospital of Southern Medical University (The First People’s Hospital of Shunde), 528300 Foshan, Guangdong PR China; 3 Departments of Hematology, Shunde Hospital of Southern Medical University (The First People’s Hospital of Shunde), 528300 Foshan, Guangdong China; 4 Medical Research Center, Shunde Hospital of Southern Medical University (The First People’s Hospital of Shunde), 528300 Foshan, Guangdong China

**Keywords:** Ferroptosis, NADPH, MARCHF6, Ubiquitination

## Abstract

Ferroptosis is a new form of programmed cell death, which achieved great breakthroughs in cell biology during past decade. However, the regulation of ferroptosis is yet to be identified thoroughly. The latest study published on *Nature cell biology* by Nguyen and colleagues found a new NADPH sensor, MARCHF6 an E3 ubiquitin ligase, mediates ferroptosis in tumor growth and animal development. This finding provides a novel insight into ubiquitin system and energy metabolism in regulation of ferroptosis, which may open up new avenues for tumor treatment.

## Commentary

Ferroptosis, a newly discovered mechanistically and morphologically different from other forms of programmed cell death with iron-dependent lipid peroxidation, is involved in various physiological and pathological processes including tumorigenesis, organ injury, autoimmune disease, and neurodegenerative disease [1, 2]. During the past decade, this new realm of cell death has attracted numerous researchers to unravel the biological processes, elucidate the molecular regulating mechanisms and yield breakthroughs in cell biology, especially molecular mechanisms and regulatory networks of ferroptosis in tumour suppression and immune surveillance [3]. Nevertheless, the regulation of ferroptosis is yet to be identified thoroughly. Previous studies demonstrated that nicotinamide adenine dinucleotide phosphate (NADPH) regulates cell ferroptosis in mammalian cells [4]. However, how NADPH regulates ferroptosis has not been well-elucidated.

A recent study by Nguyen et al. [5] published in the journal *Nature cell biology* showed that MARCHF6, an E3 ubiquitin ligase embedded in the membranes of endoplasmic reticulum with N- and C-terminal, acts as a cytosol NADPH sensor and regulates cell ferroptosis. The authors provided compelling evidence demonstrating that the intracellular content of NADPH could change the conformation of MARCHF6. This new information builds a bridge between the ubiquitin system and energy metabolism as well as offers a fundamental insight into the molecular mechanisms regulating cell ferroptosis.

The MARCHF6 not only promotes tumorigenesis and development [6] but also regulates cholesterol homeostasis [7]. Nguyen et al. [5] subjected a series of experimental manoeuvres including reduction or deletion of expression of MARCHF6 in multiple tumour cell lines and identified that this treatment increases lipid peroxidation. To understand which subunit of MARCHF6 functions as an antioxidant, the authors used either ectopic expression of C-terminally triple flag-tagged wild-type MARCHF6_3f_ or catalytically inactive mutant MARCHF6C9A 3f to treat the *MARCHF6*-knockout (KO) cells. They observed that only MARCHF6_3f_ treatment reduces the levels of lipid peroxidation; this confirms that the MARCHF6_3f_ functions as an antioxidant with the ability to suppress lipid peroxidation. Excessive lipid peroxidation is one of the hallmarks of ferroptosis [8]. Therefore, Nguyen et al. [5] exposed the *MARCHF6*-KO cells to inducers of ferroptosis (erastin and RSL3), which resulted in reducing the cell viability and promoting cell death. Importantly, the cell viability was rescued after treatment with MARCHF6_3f_ or ferroptosis inhibitors (deferoxamine or ferrostatin-1), but not with non-ferroptosis inhibitors. Moreover, overexpression of MARCHF6_3f_ also increased the viability of wild-type cells by resisting the inducers of ferroptosis. Altogether, these results reveal that MARCHF6 is involved in lipid peroxidation and ferroptosis.

To investigate how MARCHF6 affects ferroptosis, Nguyen et al. [5] implemented various approaches such as pharmacological ferroptosis inducers, metal ion additives or chelators in the genetic ablation of MARCHF6 or wild-type cells. They observed that ferric ammonium citrate (which increases intracellular Fe^2+^ content) or ferroptosis inducers increase the levels of MARCHF6 substrates but without any change in the mRNA levels of those substrates [5]. These results suggest that, during cell ferroptosis, MARCHF6 could mediate the ubiquitination of its substrates. As MARCHF6 plays a role in lipid metabolism [9], Nguyen et al. [5] also performed RNA sequencing (RNA-seq) analysis in wild-type and *MARCHF6-KO* cells to explore the molecular mechanism behind the regulation of lipid metabolism. Interestingly, MARCHF6 deletion significantly down-regulates the expression of both NADPH and the genes related to the production of NADPH in HeLa cells. Moreover, when *MARCHF6-KO* cells were treated with MARCHF6_3f_, the levels of NADPH recovered. This demonstrates that MARCHF6 regulates the levels of NADP(H) and NADPH in lipid metabolism. Additionally, overexpression of the NADK kinase (NADP(H) rate-limiting enzyme) increased the levels of NADP(H) in both wild-type and *MARCHF6-KO* HeLa cells [5]. However, overexpression of the NADK kinase resisted the cell death caused by ferroptosis inducers only in wild-type HeLa cells, but not in *MARCHF6-KO* cells. Also, the knockdown or downregulation of NADK kinase reduced the levels of NADPH and increased the levels of MARCHF6 substrates but without altering their mRNA levels, thereby inhibiting the polyubiquitylated degradation of MARCHF6 substrates. These results indicate that NADPH is indispensable for MARCHF6 ubiquitination activity and reinforce the idea that MARCHF6 regulates ubiquitin-dependent degradation of substrates during cell ferroptosis.

**Fig. 1:**
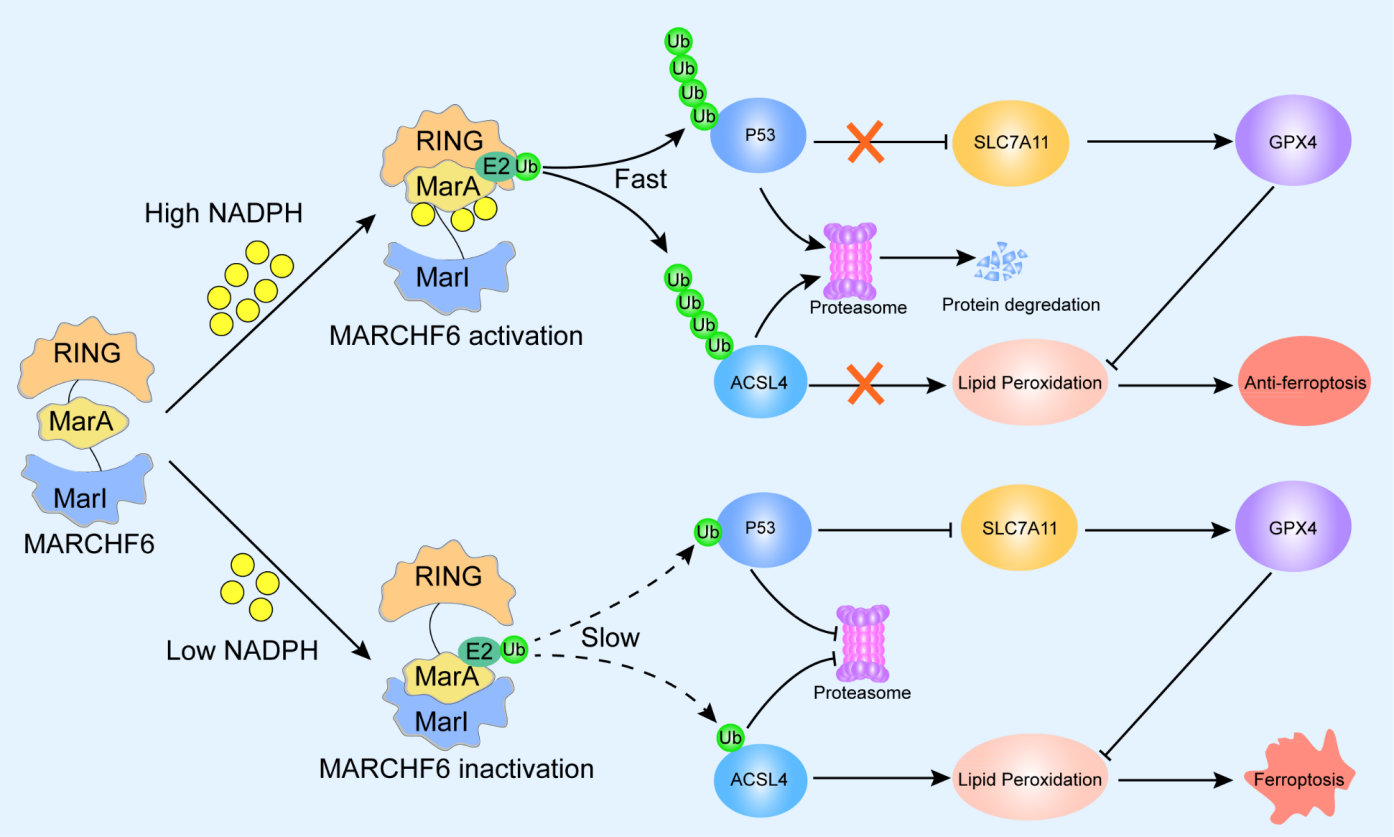
Proposed mechanism of MARCHF6 monitoring the levels of intracellular NADPH and regulating cell ferroptosis. When the intracellular levels of NADPH are high, the NADPH will directly bind with MarA, and interaction with RING thereby preventing the interaction between MarI and MarA, results in increasing the ubiquitin ligase activity of MARCHF6, promoting the ubiquitin-dependent rapid degradation of p53 and ACSL4, increasing the expression of GPX4 and SLC7A11 and decreasing the lipid peroxidation, resulting in anti-ferroptosis. When the intracellular levels of NADPH are low, the MarI binds with MarA thereby blocking the interaction of RING with MarA. This results in reducing the ubiquitin ligase activity of MARCHF6, blocking the ubiquitin-dependent degradation of p53 and ACSL4, inhibiting the expression of GPX4 and SLC7A11 and increasing the lipid peroxidation, resulting in ferroptosis

To further examine the role of NADPH in regulating the ubiquitination activity of MARCHF6, Nguyen et al. [5] predicted a three-dimensional structure of the MARCHF6 protein by AlphaFold. Subsequently, they conducted a series of experiments to explore the function of the intramolecular structure of MARCHF6. They found that the N-terminal RING domain, the C-terminal MARCHF6 inhibitory (MarI) region and the MARCHF6 activation (MarA) region are the crucial segments which regulate the activity of MARCHF6 and the intramolecular interactions in MARCHF6, based on the intracellular levels of NADPH during ferroptosis. Intriguingly, when the intracellular levels of NADPH are high, the NADPH directly binds to the MarA motif, thereby preventing the MarI from binding the MarA which promotes the E2-bound MarA with RING intramolecular interaction. This process promotes the ubiquitin ligase activity of MARCHF6 and directly mediates the degradation of substrates, pro-ferroptosis effectors (p53 and ACSL4). This further promotes the expression of anti-ferroptosis proteins such as soluble carrier family 7 member 11 (SLC7A11), glutathione peroxidase 4 (GPX4) and nuclear factor erythroid-2-related factor 2 (NRF2), thereby inhibiting ferroptosi [5] (Fig. 1). Conversely, when the intracellular levels of NADPH are low, the NADPH binding the MarA motif are reduced, the intramolecular interaction between MarI and E2-bound MarA are promoted, blocking the RING interaction with MarA. This process inhibits the ubiquitin ligase activity of MARCHF6 and suppresses the degradation of its substrates, for instance p53 and ACSL4. This also inhibits the expression of anti-ferroptosis proteins such as SLC7A11, GPX4 and NRF2, thereby promoting ferroptosis (Fig. 1). These findings demonstrate that based on the intramolecular interactions in MARCHF6, it could act as a smart sensor for cellular NADPH content and as a switcher for ubiquitin-mediated ferroptosis. Similarly, our latest study elucidated that SHARPIN, a ubiquitin-binding and ubiquitin-like domain-containing protein, regulates the expression of p53 through ubiquitination and affects the levels of SLC7A11 and GPX4 by regulating ferroptosis in cholangiocarcinoma cells [10]. Whether the ubiquitination pathway is a universal phenomenon during cell ferroptosis is an interesting idea that needs to be further explored.

To extend the relevance of these findings to in vivo settings, Nguyen et al. [5] used two mice models to analyse the function of MARCHF6. Firstly, they analysed the role of MARCHF6 in tumour progression via a xenograft mice model. The tumour size formation of MARCHF6 ablation cells is significantly smaller than the wild-type cells, and the inhibitory effect is reversed with ferroptosis inhibitor and lipid reactive oxygen species (ROS) scavenger. Secondly, they demonstrated that the MARCHF6 ablation in mice contributes to perinatal death, severe hepatic injury, growth retardation, postnatal lethality, and reduction in the birth rates. However, when the pregnant mice with MARCHF6 ablation were supplemented with a high-lipophilic antioxidant vitamin E diet or ferroptosis inhibitor, these supplements ameliorated the negative birth rates and foetal development [5]. These results signify that MARCHF6 can promote tumour growth as well as animal growth and development via the anti-ferroptosis effect.

Altogether, these novel findings strengthen our knowledge about the intramolecular interaction between MARCHF6 and NADPH as well as the function of MARCHF6 in tumour growth and animal development. We outline a few exciting avenues for future research based on these findings. Firstly, beyond the reported NADPH sensors, future research should unravel any undiscovered sensors that could monitor intracellular nutrient and metabolite levels and regulate physiological functions in cells. Secondly, the involvement of MARCHF6 in critical processes apart from cell ferroptosis and organism development should be explored. Thirdly, as the MARCHF6 ablation inhibits tumour growth and mice development, whether MARCHF6 can be a specific target for exploring drugs needs to be investigated. By exploring these avenues, novel insights may be developed regarding the role of MARCHF6 in physiological and pathological processes.

## Data Availability

Not applicable.
